# Did Usage of Mental Health Apps Change during COVID-19? A Comparative Study Based on an Objective Recording of Usage Data and Demographics

**DOI:** 10.3390/life12081266

**Published:** 2022-08-19

**Authors:** Maryam Aziz, Aiman Erbad, Mohamed Basel Almourad, Majid Altuwairiqi, John McAlaney, Raian Ali

**Affiliations:** 1 College of Science and Engineering, Hamad Bin Khalifa University, Doha P.O. Box 5825, Qatar; 2 College of Technological Innovation, Zayed University, Dubai P.O. Box 144534, United Arab Emirates; 3 College of Computer and Information Technology, University of Taif, Taif 21974, Saudi Arabia; 4 Faculty of Science and Technology, Bournemouth University, Bournemouth BH12 5BB, UK

**Keywords:** mental health, COVID-19, mindfulness, digital health, mobile health, social isolation

## Abstract

This paper aims to objectively compare the use of mental health apps between the pre-COVID-19 and during COVID-19 periods and to study differences amongst the users of these apps based on age and gender. The study utilizes a dataset collected through a smartphone app that objectively records the users’ sessions. The dataset was analyzed to identify users of mental health apps (38 users of mental health apps pre-COVID-19 and 81 users during COVID-19) and to calculate the following usage metrics; the daily average use time, the average session time, the average number of launches, and the number of usage days. The mental health apps were classified into two categories: guidance-based and tracking-based apps. The results include the increased number of users of mental health apps during the COVID-19 period as compared to pre-COVID-19. Adults (aged 24 and above), compared to emerging adults (aged 15–24 years), were found to have a higher usage of overall mental health apps and guidance-based mental health apps. Furthermore, during the COVID-19 pandemic, males were found to be more likely to launch overall mental health apps and guidance-based mental health apps compared to females. The findings from this paper suggest that despite the increased usage of mental health apps amongst males and adults, user engagement with mental health apps remained minimal. This suggests the need for these apps to work towards improved user engagement and retention.

## 1. Introduction

The World Health Organization (WHO) reports that as of 2017, there has been a 13% increase in mental health issues worldwide [1]. Mental health has been further affected since restrictions on social gatherings were placed to counter COVID-19 outbreaks [2]. The ramifications of COVID-19 include social isolation, economic crises, and unemployment, which are among the known risk factors for mental health illnesses [3]. Statista, a major provider of market and consumer data, surveyed around 23,000 participants worldwide, within the age range of 16–74 years, on mental health during the pandemic. The organization found that as of early 2021, 40% of the participants had reported a negative impact on their mental well-being during the last six months [4].

With the pandemic spreading, the interest in digital health for mental illnesses has accelerated [5,6]. Several studies, based on the populations of China [7], Spain [8], Canada [9], and Australia [10], discussed the adoption of mental health apps as a way to provide mental healthcare to their population. Along with this, there has been an increase in the number of apps claiming to provide mental health care in the market, as well as a boost in the number of downloads of these mental health apps [10,11]. The growing number of mental health app downloads could mean that more people are seeking mental health support or are receptive to it. Several demographic factors may need to be considered to identify these people, such as whether a certain age group or gender is predominantly downloading these apps. Additionally, downloading apps alone does not mean that a user is committing to use them; it would, however, show their receptiveness to help.

Jaworski et al. [12] investigated the daily usage of COVID Coach, a publicly available mental health app, with respect to the number of days the app was used. The study found that almost 50,000 people used the COVID Coach app from March 2020 to October 2020, and the app had a consistent daily active usage. Kozlov et al. [13] studied the usage of Mindfulness Coach based on the number of downloads and number of days. Their study found that the app is used infrequently and for short sessions. Almost 40% of returning users would open the app but not use it. Research conducted during COVID-19 from March to April 2020 focused on estimating the usage of popular mental health apps [14]. The study used monthly active users as a base metric to estimate usage and focused on the during-COVID-19 times. Wang et al. [15] found the number of downloads of mental health apps to increase during the COVID-19 period as compared to pre-COVID-19. Their research focused on the popular mental health apps based on the number of downloads and classified apps as per the developer’s choice.

With respect to demographics, Mackenzie et al. [16] studied the differences in help-seeking outlooks amongst the different age and gender groups using a sample size of 206 participants. Their study used questionnaires to measure the help-seeking attitudes and the mental health conditions of the participants. They found older adults above 60 and females to be more receptive to seeking help regarding their mental health. Additionally, Segal et al. [17] performed a cross-sectional study on the beliefs and help-seeking attitudes of people based on their ages. They used questionnaires to gather information about the beliefs and help-seeking attitudes of people. Their results showed that older adults aged 60–95 years reported willingness to seek help on the same level as the adults aged 17–26 years. Furthermore, Kern et al. [18] conducted a study at the university level to investigate the help-seeking attitudes of young adults aged around 18–22 years old. Their survey responses showed that most students have a positive attitude towards receiving mental health support through mental health apps. Forbes et al. [19], on the other hand, found that their survey responses showed older adults above 60 as less likely to recognize their need for mental health care.

A major limitation in the research literature relates to the utilization of self-reported data to quantify the usage of mental health apps. This may lead to reporting bias which means that participants may underestimate or overestimate their mental health behaviors. Additionally, with regard to the use of smartphone apps, such as average screen time and average launches, users tend to underestimate their smartphone usage behavior [20,21]. Furthermore, previous research studies have mostly utilized usage metrics that do not take into consideration the actual time spent on mental health apps and focus only on the number of downloads or number of days of use of mental health apps. Torous et al. [22] state that almost 70% of users leave a health app after 10 uses. The number of downloads and the number of days alone are, therefore, not representative of the usage of mental health app users. Additionally, past research investigated the popular mental health apps downloaded by users to study their usage, whereas the change in usage may only be to certain categories of these apps. For example, some apps require spending time on them, whether to meditate or do mindfulness exercises, while others are based on reminders or recording daily moods and habits.

In this paper, we compare mental health app usage before and during the COVID-19 pandemic, using objective data collected from a smartphone app that monitors smartphone usage. We analyze the users who are using these mental health apps based on age and gender. We also classify the mental health apps into two categories to study the change in usage that may exist due to the type of app. Hence, taking into consideration the pre-COVID-19 and during-COVID-19 use of mental health apps, our research questions (RQ) are:

RQ1. Is there a change in the number of users?

RQ2. Is there a change in the usage with respect to daily average time spent, the daily average number of launches, average session time, and the number of days of use?

RQ3. Is there a change in the usage across the two categories of mental health apps: the guidance-based and the tracking-based apps?

## 2. Methods

### 2.1. Dataset

The dataset for this research was collected through an app that helps users monitor their phone usage by tracking their usage, such as phone unlocks, launches, and sessions of each app they use. It also collects information related to user demographics, including age and gender. The app privacy policy, to which all users consent, includes that collected data can be shared with academic partners for research purposes. Nevertheless, the app required the explicit consent of users before collecting and utilizing their data for this research. 

Data collection for this study was first conducted in 2019 and then repeated in 2020 during COVID-19. The pre-COVID-19 data collection took place between June 2019 and September 2019. The purpose of that data collection was to study the relationship between smartphone usage and certain psychometrics of the user collected through a questionnaire they answered. The data collection during the COVID-19 period was conducted from October 2020 to April 2021. Throughout the paper, pre-COVID-19 is referred to interchangeably with 2019, while during-COVID-19 is referred to as 2020. The data collection was open to new participants who installed the app throughout the study duration, and our participants joined and withdrew from the app after a different number of days. The study was conducted over a period of 21 days from the start date of each user. The 21 days period choice covered three weeks, including weekends, and included a considerable number of users since some left the study after that. 

The 2019 dataset had 376 users, while for the 2020 dataset, 557 users participated. Users in the 2020 study came from ten countries: Sweden, Australia, Netherlands, Canada, Germany, India, the United Kingdom, Brazil, France, and the United States. We wanted to restrict the study to countries where restrictions on social gatherings were applied. The 2019 dataset had around 70% users from the same 10 countries as the during-COVID-19 dataset. The datasets included anyone who participated, even for one day. Furthermore, the dataset included only Android smartphone users.

### 2.2. Data Preparation

The pre-processing of the data was conducted using the programming language Python 3.0 [23]. The dataset received had a total of 1070 users from the pre-COVID-19 and during-COVID-19 periods. However, a few users had chosen not to enter their age and gender, bringing the number of users taken for this study to 933. The parameter “Age” was categorized into five different categories: 15–24, 25–34, 35–44, 45–54, and 55–64. Based on the UNICEF age categorization [24], age was categorized into two categories of emerging adults (15–24 years) and adults (above 24). This categorization also helped to balance the dataset, given that the number of participants in categories above 15–24 was comparatively less. 

Furthermore, the third-party app records each app session for each user, i.e., the app name and the timestamps of the start and end of use. The records of app sessions were thoroughly checked and cleaned to remove duplicates and anomalies and concatenate broken down sessions. Table 1 presents a sample of the data collected from the third-party app. From the app sessions, the time spent on each app was calculated in seconds. Additionally, each session was considered as a launch of an app. 

The raw data collected included only the name of the apps used but not the category of these apps, that is, whether they are in the social media, communications, or gaming category. Therefore, we extracted the app categories and descriptions based on the Google Play Store, using software utilizing Google Play API. Table 2 shows a sample of the app descriptions we received from Google Play API. Google Play Store groups apps into 49 categories, including health and fitness and medical categories [25]. App categorization is based on the developer’s choice; therefore, a few apps were categorized incorrectly. Due to this, we took the apps in the health and fitness and the medical app categories as well as the top apps used in both productivity and lifestyle categories. Additionally, to ensure all health-related apps were extracted from other categories, we searched for keywords such as “health”, “fitness”, “medical”, and “disease” in the description of the apps. Health and fitness apps contain apps related to personal fitness, workout tracking, health, and safety, while medical apps contain apps related to clinical references, clinical apps, and medical journals, amongst others. For this study, after we extracted the health and medical apps, we then manually checked these extracted apps to find the mental health apps. 

Around 800 apps were identified using the above method, out of which 690 were health and medical-related. Out of these 690 apps, 115 mental health-related apps were found. The apps classified as mental health covered various areas, including online therapy, mindfulness, meditation, and well-being. For example, the apps that met the inclusion criteria included I am Sober [26], Wysa [27], Happify [28], Headspace [29], and Calm [30]. Apps such as those focused primarily on yoga and healthy living were removed since these were more directed towards lifestyle than mental health. 

The National Institute of Mental Health categorizes mental health apps into six categories: self-management apps, apps for improving thinking skills, skill-training apps, social support apps, passive symptom tracking apps, and data collection apps [31]. These six categories of apps can be grouped into two categories based on the time and frequency of use, which are the focuses of this study. The classification of the subcategories of the mental health apps was achieved by first coding the mental health apps based on whether they were for online therapy, mental health support, meditation exercises, mindfulness, or tracking. We then marked the apps for online therapy, mental health support, meditation exercises, and mindfulness as time-based apps since users need to spend time on these apps to meet the purpose for which the app is developed. We marked the tracking apps as frequency-based apps since users typically need to launch these apps frequently to meet the purpose. As such, apps that require a user to spend some time on them can then be called guidance-based since they mostly guide users towards developing coping and cognitive skills through mindfulness exercises, meditation, online therapy, and host support groups, among other mental well-being tools. Examples of guidance-based apps are Headspace, Calm, and Wysa. On the other hand, apps that require the user to launch them occasionally but not spend a long time on them can be referred to as tracking-based apps since these mostly include mood-tracking, symptoms tracking, and addiction-tracking apps. Examples of such apps include I am Sober and Anxiety Tracker [32]. The mental health apps were categorized according to these two categories of guidance-based and tracking-based apps. Since a few mental health apps belonged to both subcategories, the apps’ primary focus was used when categorizing them as either a guidance-based app or a tracking-based app. There was a total of 36 tracking-based apps, while the guidance-based apps totaled 79 apps.

### 2.3. Data Preparation

The usage of mental health apps for each participant was measured through four different metrics, which are the average daily time spent on mental health apps (DT), the average daily number of launches of mental health apps (DL), the average duration of daily sessions of mental health apps (DS), and the number of days of use of mental health apps (UD) during the 21 days period.

The average daily time spent was measured by finding the total daily time spent, in minutes with fractions of seconds, on the apps over the 21 days. For the average daily launches of the apps, the total daily count of sessions on the apps was taken over 21 days. Additionally, the average duration of daily sessions was calculated by taking the sum of the sessions throughout the 21 days and averaging them over 21. The number of days of use was calculated as a count of the unique days of the usage of mental health apps. The four usage metrics were calculated separately for the overall mental health apps, guidance-based mental health apps, and tracking-based mental health apps.

The study was designed to answer three different research questions and, therefore, required different criteria for the usage metrics. The first question (RQ1) was directed toward the number of users of mental health apps before and during COVID-19 and did not require the utilization of the four usage metrics (DT, DL, DS, and UD). The second question (RQ2) focused on answering the change in the usage of the overall mental health apps before and during COVID-19. In this case, all four usage metrics were measured for the participants. Additionally, we had 11 users in 2019 and 14 users in 2020 with fewer than 2 days of usage. We also had 327 users in 2019 and 452 users in 2020 with no usage. The users with no usage or number of days of use fewer than 2 days over the 21 days were not considered. This was undertaken to take into consideration that users with fewer than 2 days of usage may have installed mental health apps as a trial and hence, may not have returned to use them. Furthermore, they had a negligible time spent on mental health apps (below 2 min). The third question (RQ3) dealt with the change in usage of the two categories of mental health apps before and during COVID-19. We took all four usage metrics in this case as well and did not consider users with no usage or usage fewer than 2 days. We found 11 users in 2019 and 14 users in 2020 with fewer than 2 days of usage for guidance-based apps. Meanwhile, for tracking-based apps, we had 2 users in 2019 and 5 users in 2020. With regards to users having no usage, we had 335 users in 2019 and 478 users in 2020 for guidance-based apps. For tracking-based apps, we had 359 users with no usage in 2019 and 531 users in 2020. Similar to RQ2, the users with fewer than 2 days of usage were not considered since they had negligible time spent on mental health apps and may have installed the mental health apps as a trial.

### 2.4. Data Analysis

The statistical analysis was performed on JASP 0.14.1 [33]. Chi-square tests were used to determine the change in the number of users from pre-COVID-19 to during COVID-19. Chi-square tests were also further used to determine the relationship between using mental health apps and the demographic variables of their users. Phi-coefficients were used as well to determine the effect size of these relationships. The normality of the data was checked by conducting Shapiro–Wilk tests on the four usage metrics (DT, DL, DS, and UD) with respect to the years and demographics for the overall mental health, guidance-based, and tracking-based apps. The majority of the usage metrics (that is, 66 out of the 96 distributions) did not have a normal distribution; hence, non-parametric tests were considered. Median and interquartile ranges (IQR) were used for the descriptive statistics since the usage measures were not normally distributed. Since the usage metrics were continuous variables, the Mann–Whitney U test was further applied to compare the usage from pre-COVID-19 to during COVID-19. Mann-Whitney U test was first conducted separately against the usage and the time periods for overall mental health apps, guidance-based and tracking-based apps. Then, the usage was tested against age and gender for the overall mental health apps, guidance-based, and tracking-based apps. Cohen’s d was used to determine the effect size of these relationships.

## 3. Results

### 3.1. Descriptive Statistics

The first question (RQ1) is concerned with whether the number of mental health app users before and during COVID-19 is significantly different. For RQ1, we had a sample size of 376 participants in 2019, of which around 47% were females and around 55% were emerging adults. For the 2020 dataset, the sample size had 557 participants, of which 58% were females and 50% were emerging adults.

The second question (RQ2) is concerned with the change in the usage of overall mental health apps before and during COVID-19. For RQ2, we had a sample size of 38 participants in 2019, of which around 32% were females and around 30% were emerging adults. The 2020 dataset for RQ2 had a sample size of 81 participants, of which around 63% were females and around 40% were emerging adults. The sample size was reduced compared to RQ1 because of the exclusion of users with no usage or usage less than 2 days, as mentioned previously. Table 3 shows the descriptive statistics of the usage of mental health apps in 2019 and 2020.

The third question (RQ3) is concerned with the change in the usage of guidance-based and tracking-based apps before and during COVID-19. For RQ3, we had a sample size of 30 participants in 2019 for guidance-based apps. Of the 30 participants, around 37% were females and around 27% were emerging adults. For the 2020 dataset, the sample size was 65 participants, of which around 62% were females and around 38% were emerging adults for guidance-based apps. Table 4 shows the descriptive statistics of the usage of guidance-based mental health apps in 2019 and 2020. For the tracking-based mental health apps, the 2019 dataset had a sample size of 15 participants, of which around 13% were females and around 47% were emerging adults. For the 2020 dataset, the sample size had 21 participants, of which around 76% were females and around 52% were emerging adults. Table 5 shows the descriptive statistics of the usage of the tracking-based mental health apps in 2019 and 2020. The categories of guidance-based and tracking-based mental health apps were taken independently; hence some users had usage for both guidance-based and tracking-based mental health apps and were considered participants of both these categories. 

**Figure 1:**
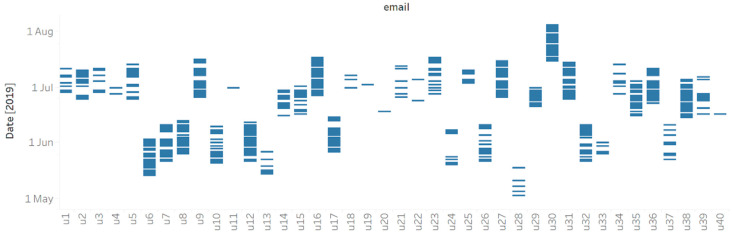
Usage sparsity for a sample of 40 users from 2019.

**Figure 2:**
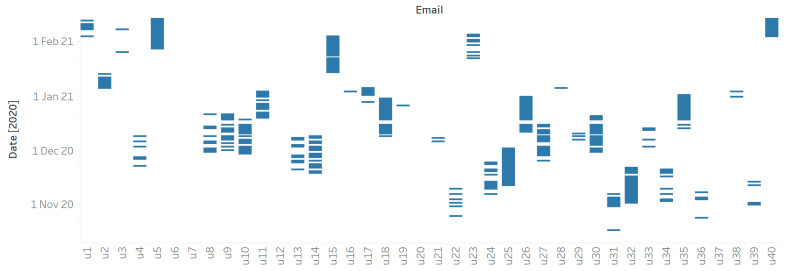
Usage sparsity for a sample of 40 users from 2020.

With respect to the days of use, the users did not use the mental health apps regularly and had sparse usage, showing that they left for some days before returning to use the mental health apps. In the 2020 dataset, around 49% of the users had a usage of less than or equal to 7 days, while around 31% had a usage of around 21 days. On the other hand, for the 2019 dataset, 39% of the users had a usage of less than or equal to 7 days, while 37% of users had a usage of around 21 days. Figure 1 represents this sparsity in the usage of the users in 2019 for a sample of 40 random users. Similarly, Figure 2 represents the sparsity in the usage of the users in 2020 for a sample of 40 random users. The days of use for the 40 users in both years are marked. The visualization shows a general lack of consistent and durable usage. For example, u4 and u11 in 2019 used such apps for only a few days in early July. 

### 3.2. App Usage

#### 3.2.1. RQ1: Changes in the Number of Mental Health Apps Users

We first compared the number of mental health app users between 2019 and 2020. The results, *χ*^2^(df = 1, N = 933) = 4.77, *p* = 0.029, show that the relationship is significant with an effect size, w = 0.071. Compared to 2019, the number of users increased in 2020. Furthermore, when comparing the years with age in 2019, the results, *χ*^2^(df = 1, N = 376) = 15.86, *p <* 0.001, showed the relationship to be significant, with an effect size of w = 0.21. Adults were more likely to be users of mental health apps compared to emerging adults. There was no significant difference between the change in the number of adult and emerging adult users in 2020. No significant relationship was found between gender and mental health app users, whether within 2019 or 2020.

#### 3.2.2. RQ2: Changes in Usage Time, Launches, Session Time and Number of Days

The results showed no statistically significant difference between the average daily usage time of mental health apps between 2019 and 2020. Additionally, no statistically significant relationship was found between mental health app usage and the demographics within 2019 and 2020. The results from comparing the change in mental health usage based on average session time also had no statistically significant relationship within and across 2019 and 2020. 

The daily average number of launches of mental health apps was found to be statistically significant for gender in 2020. The average number of launches was greater for males (Mdn = 1.43, n = 30) compared to females (Mdn = 0.76, n = 51), *U* = 982, *p* = 0.034, |*r*| = 0.28.

For the number of days of use, age, and gender were both significant for 2020. The results showed that males (Mdn = 13.0 days, n = 30) had a higher number of days of use compared to females (Mdn = 7.0 days, n = 51), *U* = 994.5, *p* = 0.025, |*r*| = 0.30. On the other hand, in terms of days of use, adults (Mdn = 10.5 days, n = 48) were more likely to use mental health apps compared to emerging adults (Mdn = 5.0 days, n = 33), *U* = 547, *p* = 0.018, |*r*| = 0.31.

#### 3.2.3. RQ3: Changes in Usage across the Two Categories of Mental Health Apps 

The results for the daily average time spent on guidance-based mental health apps were found to have no significance with the period, age, and gender. Comparing the average session time for guidance-based apps with the year 2019 and 2020 showed a significant relationship where users in 2020 (Mdn = 1.34 min, n = 65) had a higher daily average session time compared to users in 2019 (Mdn = 1.05 min, n = 30), *U* = 695, *p* = 0.025, |*r*| = 0.29.

The results for the daily average number of launches of guidance-based mental health apps showed age to be significant for 2019 and both age and gender to be significant for 2020. For 2019, the results showed emerging adults (Mdn = 0.38, n = 8) as having lower daily average launches compared to adults (Mdn = 1.60, n = 22), *U* = 39, *p* = 0.023, |*r*| = 0.56. Similarly, in 2020, emerging adults (Mdn = 0.48, n = 25) were found to have lower daily average launches compared to adults (Mdn = 1.60, n = 40), *U* = 288.5, *p* = 0.004, |*r*| = 0.42. Additionally, males (Mdn = 1.48, n = 25) were found to have higher number of daily average launches compared to females (Mdn = 0.74, n = 40), *U* = 649, *p* = 0.045, |*r*| = 0.30.

The number of days of use of guidance-based apps showed significant results for age in both 2019 and 2020. In 2019, adults (Mdn = 12 days, n = 22) had a higher number of usage days of guidance-based mental health apps compared to emerging adults (Mdn = 3.5 days, n = 8), *U*= 32, *p* = 0.009, |*r*| = 0.64. Similarly, in 2020, adults (Mdn = 11.5 days, n = 40) again showed a higher number of usage days for guidance-based mental health apps compared to emerging adults (Mdn = 4 days, n = 25), *U* = 247.5, *p* < 0.001, |*r*| = 0.51. 

For tracking-based mental health apps, a significant relationship for gender was found in 2020 where males (Mdn = 17 days, n = 5) had a higher usage in terms of number of days compared to females (Mdn = 6 days, n = 16), *U* = 68.5, *p* = 0.020, |*r*| = 0.712.

## 4. Discussion

This study compares the use of mental health apps before and during the pandemic amongst different age and gender groups. There are compelling reasons to directly focus on this area of study since, with remote lifestyle becoming the norm, digital tools are being used increasingly to provide guided and unguided mental health care remotely [34]. The findings from this paper are objective, as the data were used to accurately quantify the mental health app usage of users from 10 different countries, as compared to the use of self-reported data in the literature. Additionally, these findings can also be applied to a social isolation setting since limiting the spread of COVID-19 has resulted in prolonged periods of social isolation. Being socially isolated for even less than 10 days can cause long-term mental health problems [35]. Table 6 summarizes the findings related to RQ1, and Table 7 summarizes the findings related to RQ2 and RQ3.

### 4.1. Mental Health Apps and Subcategories with Respect to Number of Users

The findings from this study show that there was a significant increase in the number of mental health app users from 2019 to 2020. This is in line with the report from ORCHA [11], stating that the number of downloads of mental health apps has increased during the pandemic. This increase in downloads of mental health apps could be attributed to the social isolation that has come into play due to the pandemic. This is supported by a study conducted by Chan and Honey [36] to understand user perceptions of mental health apps. They stated that although face-to-face mental health support cannot be replaced, mental health apps have the potential to be an add-on source for some and an alternative option for others to receive mental health support. With respect to guidance-based apps, the average session time also increased in 2020 compared to 2019. With mindfulness, meditation, online therapy, and other mental well-being exercises being a part of guidance-based apps, the increased average session time could be explained by users shifting their mental health care and needs to digital platforms. 

### 4.2. Mental Health Apps and Subcategories with Respect to Gender

Overall mental health app usage showed an interesting outcome of gender being significant in 2020 with respect to the daily average number of launches and number of days of use of mental health apps. Moreover, in studying the guidance-based apps, gender was also significant in 2020 with respect to the daily average number of launches. For tracking-based apps, gender was again significant in 2020 with respect to the number of days of use. In all cases, males were found to be higher users compared to females. The previous research that was undertaken in this domain show males as having a more negative attitude when it comes to mental health treatment and hence, not receiving the care they need [37]. However, previous research undertaken in this domain mostly used self-reporting, and the participants may have not properly assessed themselves. The current study is conducted using objective data to measure the usage of mental health apps and identify the users of these apps. Therefore, the current results show male and female users to have similar usage pre-pandemic but increased usage by males during the pandemic. When using a self-reporting data collection method, e.g., surveys and interviews, whether for research or counselling purposes, females are more open when declaring their mental health help-seeking behavior as compared to males [16]. However, when using the objective measures of usage of mental health apps, both males and females had similar usage in 2019 and, hence, similar help-seeking behaviors. Further, in 2020, males had comparatively higher usage of mental health apps, which may suggest they are more likely to seek support for their mental health using digital means. This may relate to the stigma associated with seeking help when it relates to mental health, which is more prominent in males [38], and the tendency of males to prefer more anonymous options than females. In other words, our results suggest that while males are less likely than females to seek help through traditional means of therapy and health institutions, they are more open to adopting digital means for that same purpose.

### 4.3. Mental Health Apps and Subcategories with Respect to Age

The results also showed age to be significant in 2019 with respect to the number of users; that is, adults are more likely to be users of mental health apps compared to emerging adults. The during-pandemic period also showed adults using mental health apps more as opposed to emerging adults with respect to average daily launches and number of days of use of mental health apps. In addition, when dividing mental health apps into categories of guidance-based and tracking-based, both the 2019 and 2020 datasets showed that the daily average number of launches and number of days of use for guidance-based apps was comparatively higher for adults than emerging adults. This means that whether the period was pre-pandemic or during-pandemic, adults’ usage of mental health apps is higher than emerging adults. These findings align with the past research of Mackenzie et al. [16], which found that adults are highly likely to seek help regarding their mental health compared to emerging adults. Moreover, this also shows that, regardless of a crisis, emerging adults are not likely to seek mental health support, despite having access to them. The research undertaken by Kern et al. [18] showed that although young adults were interested in adopting mental health apps, their usage of these apps was limited. In general, mobile health apps attract young adults due to the immediate access provided by them; however, young adults tend to abandon them, citing costs and user experience as demotivating factors for continued use [39].

### 4.4. User Engagement and Retention of Mental Health Apps

The average time spent on overall, guidance-based, or tracking-based mental health apps showed no significant differences in the current study. This unchanged usage for the two time periods could be explained by the fact that the users require the mental health apps only when needed and may use them with extended breaks between usage [12]. This is also seen in the sparse usage of mental health apps in 2019 and 2020, with 37% of the users using for 21 days from their start date for 2019 and 31% of the users using for 21 days from their start date for 2020, resulting in their average time spent being unchanged during both time periods. For guidance-based apps, we would expect users to spend more time on them doing meditation exercises or mindfulness practices compared to tracking-based apps. However, the relatively similar time may suggest that the users do not follow through with the apps’ objectives. According to Kozlov et al. [13], the mindfulness app called Mindfulness Coach had returning users who would only launch the app but not use it for the mindfulness exercises. This means that these apps fail to retain users for extended periods and may need to adopt a just-in-time intervention technique [40] to increase user engagement. The intervention method would provide mental health support to users when they need it or when they ask for it. Furthermore, user engagement and retention with mental health apps can be enhanced by adopting gamification techniques, i.e., the use of game-like design elements for a meaningful purpose [41,42]. Gamification uses gaming dynamics such as rewards and levels to keep the users motivated and engaged [43]. Additionally, Chiauzzi and Newell [44] found that 23% of users leave mental health apps after 1 week, with users using a mood-tracking app as intended for no more than 2 weeks. Based on a meta-analysis of mental health interventions [45], the length of an intervention for mental health last from 4 to 16 weeks, whereas users do not use mental health apps long enough for an intervention. Privacy concerns, lack of effectiveness of the apps, absence of user-centered design, and inadequate usability standards could be the reasons behind the low engagement with mental health apps for long-term periods [22]. Despite mental health apps having a low user engagement, they show the users’ intent on seeking mental health support. The hesitation in adopting mental health apps as a steady medium of support is understandable since most mental health apps are developed without the presence of a mental health professional [46,47]. Additionally, a study on mental health apps based on clinical and scientific evidence found that only a small amount of mental health apps are based on clinical and evidence-based interventions. This means that a large number of mental health apps available on the Apple Store and Google Play Store do not go through rigorous testing within healthcare contexts to ensure the effectiveness and safety of mental health apps [48]. These mental health apps also do not remain in the market for long since they are mostly developed by small teams of developers with no future plans for support and upgrades [49].

### 4.5. Limitations

The study has its limitations with regard to the sample size used. The sample size was large enough to study mental health users, overall mental health app usage, and guidance-based mental health apps. However, for tracking-based apps, the number of users was considerably small since, despite the large sample size of 378 users in 2019 and 557 users in 2020, overall mental health apps were used by 38 users in 2019 and by 81 users in 2020. Although this number of users helped us to make a comparison between the users and non-users of mental health apps, they also limited the further analysis that could have been conducted on the users of mental health apps themselves. Another limitation in the study is the time span of 21 days taken for this study to include most users since some users left the study. As previously noted, mental health apps are utilized with extended periods of break, and as such, only when facing the need, the time span of the study may need to be lengthened to account for the periods of breaks in between. Furthermore, the third-party app collected the age of participants as an age range rather than an exact number. The utilization of an exact age could help divide age into further refined categories such as late adolescents and young adults. This would help to produce more accurate results with respect to the usage of mental health apps and age.

## 5. Conclusions

With the findings of this paper, the change in mental health app usage pre-pandemic and during-pandemic can be realized. The help-seeking behavior that was associated with females is found to be equally existing in males pre-pandemic and increased during-pandemic. In addition, it was also found that during the pandemic, adults are engaging more in mental health apps than emerging adults. This study helps in identifying the groups that are truly using these apps and those that are not since objective data were collected with regards to mental health app usage. This research shows how mental health apps are helping users through the pandemic, and hence, regulations on these apps should be enforced to ensure their safety of usage for the mental health of users. Despite the change in usage amongst users, user engagement and retention were not substantial for these apps; hence, the findings from this paper also suggest a need to adopt improved user engagement and retention techniques such as just-in-time intervention by developers of mental health apps. 

Future work in this area may focus on studying the users who used mental health apps occasionally and whether they used them intensively during these times. Another future direction of study may investigate and identify the users who left mental health apps and whether they left due to improved conditions or disinterest in the mental health apps. The type of apps these users use can also be studied to identify the categories of mental health apps that have the most dropout. Furthermore, for this study, only two demographics of age and gender were considered, while future studies may also investigate the impact of culture and education as well as personality and type of mental health issues. Future studies in this domain could also look into the usage of guidance-based apps and tracking-based apps with respect to identifying user clusters that, in fact, do utilize these subcategories as per the apps’ objectives and those that do not.

## Figures and Tables

**Table 1:** Sample of data collection from the third-party app.

User	Website	Start Time	End Time
u1	Facebook	18-January, 1:40:53	18-January, 1:42:47
u1	WhatsApp	18-January, 9:59:35	18-January, 10:00:03
u1	Happify	18-January, 10:00:03	18-January, 10:00:08
u1	Happify	18-January, 10:07:37	18-January, 10:07:38
u1	Happify	18-January, 10:28:52	18-January, 10:28:54

**Table 2:** Sample of app categories and descriptions based on Google Play Store.

Apps	Title	Category	Category Id
Gmail	Gmail	COMMUNICATION	C7
WhatsApp	WhatsApp Messenger	COMMUNICATION	C7
Tumblr	Tumblr	SOCIAL	C44
Wysa	Wysa: stress, depression & anxiety therapy chatbot	HEALTH_AND_FITNESS	C31
360 medics	360 medics	MEDICAL	C36
7 Cups	7 Cups: Anxiety & Stress Chat	HEALTH_AND_FITNESS	C31

**Table 3:** Overall mental health apps descriptive statistics.

	2019 (N = 38)				2020 (N = 81)			
	Daily Average Time spent	Daily Average Number of Launches	Average Session Time	Number of Days of Use	Daily Average Time Spent	Daily Average Number of Launches	Average Session Time	Number of Days of Use
Median	1.22	1.58	1.01	9.50	1.39	0.86	1.16	8.00
IQR	2.16	1.98	0.95	11.00	2.30	1.81	2.08	12.00

**Table 4:** Guidance-based mental health apps descriptive statistics.

	2019 (N = 30)				2020 (N = 65)			
	Daily average Time Spent	Daily Average Number of Launches	Average Session Time	Number of Days of Use	Daily Average Time Spent	Daily Average Number of Launches	Average Session Time	Number of Days of Use
Median	1.21	1.12	0.98	9.00	1.49	0.76	1.23	7.00
IQR	1.97	1.84	1.06	12.00	2.28	1.81	2.31	12.00

**Table 5:** Tracking-based mental health apps descriptive statistics.

	2019 (N = 15)				2020 (N = 21)			
	Daily Average Time Spent	Daily Average Number of Launches	Average Session Time	Number of Days of Use	Daily Average Time Spent	Daily Average Number of Launches	Average Session Time	Number of Days of Use
Median	0.63	1.00	1.14	7.00	0.58	0.95	1.01	8.00
IQR	1.26	1.48	0.73	11.00	1.65	0.95	0.99	10.00

**Table 6:** Summary of results of the analysis performed to answer RQ1.

	Number of Users					
	N (df)	*χ* ^2^	*p*			
Year	933 (1)	4.77	0.029 *			
	**2019**			**2020**		
	**n (df)**	***χ*** ^**2**^	***p***	**n (df)**	***χ*** ^**2**^	***p***
Age	376 (1)	15.86	<0.001 *	557 (1)	2.82	0.093
Gender	376 (1)	1.61	0.205	557 (1)	2.95	0.086

* *Significance level* < 0.05.

**Table 7:** Summary of results of the analysis performed to answer RQ2 and RQ3.

		Mental Health Apps					
Year		n	*U*	*p*			
		119					
Daily average time spent			1476.50	0.72			
Daily average number of launches			1741.50	0.25			
Average session time			1215.00	0.065			
Number of days of use			1711.00	0.33			
		**2019**			**2020**		
		**n**	***U***	***p***	**n**	***U***	***p***
		38			81		
Daily average time spent	Age		121.50	0.39		748.00	0.68
	Gender		119.50	0.26		910.00	0.16
Daily average number of launches	Age		111.50	0.24		625.00	0.11
	Gender		138.50	0.59		982.00	0.034 *
Average session time	Age		141.00	0.82		911.00	0.26
	Gender		125.00	0.34		769.00	0.97
Number of days of use	Age		108.00	0.20		547.00	0.018 *
	Gender		142.50	0.68		994.50	0.025 *
		**Guidance-based mental health apps**					
**Year**		**n**	***U***	***p***			
		95					
Daily average time spent			853.50	0.33			
Daily average number of launches			1037.00	0.62			
Average session time			695.00	0.025 *			
Number of days of use			1062.50	0.49			
		**2019**			**2020**		
		**n**	***U***	***p***	**n**	***U***	***p***
		30			65		
Daily average time spent	Age		46.50	0.054		382.50	0.12
	Gender		65.00	0.093		395.00	0.16
Daily average number of launches	Age		39.00	0.023 *		288.50	0.004 *
	Gender		83.00	0.37		351.00	0.045 *
Average session time	Age		78.00	0.66		581.00	0.28
	Gender		73.00	0.19		483.00	0.83
Number of days of use	Age		32.00	0.009 *		247.50	<0.001 *
	Gender		76.00	0.23		360.50	0.059
		**Tracking-based mental health apps**					
**Year**		**n**	***U***	***p***			
		36					
Daily average time spent			162.50	0.89			
Daily average number of launches			167.00	0.77			
Average session time			159.00	0.98			
Number of days of use			153.00	0.90			
		**2019**			**2020**		
		**n**	***U***	***p***	**n**	***U***	***p***
		15			21		
Daily average time spent	Age		24.00	0.69		79.00	0.098
	Gender		12.00	0.93		44.50	0.74
Daily average number of launches	Age		22.50	0.56		75.70	0.16
	Gender		10.00	0.67		63.00	0.063
Average session time	Age		32.00	0.69		73.00	0.22
	Gender		18.00	0.48		26.00	0.28
Number of days of use	Age		22.50	0.56		63.50	0.57
	Gender		11.00	0.79		68.5	0.02 *

* *Significance level* < 0.05.

## Data Availability

Derived data supporting the findings of this study are available from the corresponding author on request.
